# The socioecological correlates of collective movements in giraffes

**DOI:** 10.1093/beheco/arag098

**Published:** 2026-08-21

**Authors:** Herbert Kasozi, Micheal Mutebi, Julius Muge, Allan Kakuru, Ariana Strandburg-Peshkin, Elodie F Briefer

**Affiliations:** Department for the Ecology of Animal Societies, Max Planck Institute of Animal Behavior, Bücklestraße 5a, Konstanz 78467, Germany; Department of Zoology, Entomology and Fisheries Sciences, Makerere University, P. O. Box 7062, Kampala, Uganda; Department of Zoology, Entomology and Fisheries Sciences, Makerere University, P. O. Box 7062, Kampala, Uganda; Department of Zoology, Entomology and Fisheries Sciences, Makerere University, P. O. Box 7062, Kampala, Uganda; Department for the Ecology of Animal Societies, Max Planck Institute of Animal Behavior, Bücklestraße 5a, Konstanz 78467, Germany; Behavioural Ecology Group, Section for Ecology and Evolution, Department of Biology, University of Copenhagen, Universitetsparken 15, Copenhagen 2100, Denmark

**Keywords:** giraffe, coordination, decision-making, group movement, fission-fusion

## Abstract

How collective behaviors emerge from group members with potentially divergent needs remains poorly understood in many social species. Giraffes are known to exhibit substantial social fluidity, given the high degree of fission-fusion dynamics that shape their grouping patterns. However, the patterns and processes underlying giraffe collective movements remain unknown. Using behavioral observations, we examined how individual factors (sex and age), social context (group size and type), and habitat characteristics (open and closed) influence the decision-making process that shapes giraffe collective movements and resultant group fission. We found that adult females were more likely to initiate group movements, whereas subadults followed movements more quickly than other age classes. Decision-making for collective movements was slower in larger groups than in smaller ones. Consequently, movements in larger groups were more likely to result in group fission. These results contribute to a growing body of knowledge on the coordination of collective movement across species. By illuminating collective movement dynamics in an understudied large mammal species with a high degree of fission-fusion dynamics, we contribute to the potential for cross-species investigations into the mechanisms of collective behavior and the factors that drive inter- and intraspecific variation.

## Teaser

Giraffes often travel together to find food and water. We found that adult giraffes, especially females, were most likely to initiate group movements. Younger giraffes were quicker to follow these movements than older ones. Larger groups, particularly those in open areas, tended to split after movement initiation. These findings help us understand how giraffes coordinate their movements and how their social groups change over time.

## Introduction

Social animals interact in groups comprising related or unrelated individuals of different ages and sexes (Krause and Ruxton 2002; Kasozi and Montgomery 2020). Social interactions within animal groups may often be nonrandom, with individuals preferentially associating with particular group members, and may be driven by kinship, social bonds, predation risk, information exchange, or other benefits associated with group living (Bashaw et al. 2007; Wittemyer et al. 2008; Duboscq et al. 2016; Valentini et al. 2020). Thus, coordinating collective behavior during social interactions hinges on integrating both individual and group needs (Seppänen et al. 2007; Kasozi and Montgomery 2018; Strandburg-Peshkin et al. 2018). For instance, to maintain cohesion when moving together, group members may need to synchronize their activities (King and Sueur 2011; Seltmann et al. 2013; Ramos et al. 2015). However, coordination of collective movements may not be easily achieved when group members engage in mutually exclusive activities due to individual variation in physiological and ecological needs (eg moving to find water versus moving to find food). The varied needs of group members shape their preferences for when and where to move (Franks et al. 2002). Individuals are predicted to decide to join group movements when the perceived benefits of joining a collective movement outweigh the costs of deciding otherwise (Conradt and Roper 2005).

A common context in which coordination is required to maintain cohesion with conspecifics is the transition from being stationary to traveling. In strongly cohesive groups, the constraint of staying together during travel requires individuals to reach consensus on both the timing and direction of travel across all group members (Conradt and Roper 2005). Consensus decisions are generally thought of as ranging from “despotic/unshared”, where 1 individual (usually a socially dominant 1) dictates the outcome, to “democratic/shared”, where all group members contribute to the group decision (Conradt and Roper 2005, 2009). However, in species with more fluid social dynamics, where the constraint of staying together is relaxed or nonexistent, group members can make individual decisions that combine to form coordinated group-level outcomes (Conradt and Roper 2005). In this context, individual decisions are not independent but socially influenced by other group members. In other words, the influence spreads additively across the group. When influence spreads only among a fraction of group members, the group often splits, with individuals traveling in separate directions or only a subset moving as occurs in species with high degrees of fission-fusion dynamics (Aureli et al. 2008). The mechanistic individual decision-making processes underlying collective movements that drive and maintain fission-fusion dynamics have not been widely investigated across many species (Madsen and de Silva 2024).

Collective movements are generally structured into a predeparture period, followed by the initiation of movement by specific individuals and recruitment of followers (King and Sueur 2011). The predeparture period consists of a preparation phase during which some or all group members may express their intent to move via several behavioral cues. For instance, the intent to move may be communicated among group members via auditory or visual signaling (Watts 2000). The signaling group members may then depart and subsequently attract the attention of other group members, prompting them to follow. The decision-making process underlying collective movement in large social mammal species that associate in fission-fusion groups has been investigated more widely in primates than other taxonomic groups (eg Watts 2000; Sueur and Deneubourg 2011; Schweinfurth et al. 2022). To our knowledge, no study has investigated the decision-making process underling collective movements in giraffes (*Giraffa spp.*).

Giraffes are large, socially complex ungulates that inhabit diverse savannah ecosystems across sub-Saharan Africa (Dagg and Foster 1976; O’Connor et al. 2019). Their social system is structured by preferential and avoided associations among individuals based on sex, kinship, or age (Bercovitch and Berry 2013; Carter et al. 2013; Muller et al. 2018; Bond et al. 2021b). Adult females tend to be philopatric and maintain long-term stable associations with offspring across generations (Bercovitch et al. 2006; Carter et al. 2013; VanderWaal et al. 2014; Ferres et al. 2021; Bond et al. 2021b). By contrast, males tend to loosely associate in nonkin-based groups comprising individuals from various natal female groups (Bercovitch et al. 2006; Carter et al. 2013; Bond et al. 2021a, 2021b). However, more stable associations have been observed among males in some small populations (Prehn et al. 2019). Adult males constantly move between female groups in search of mates and may become more solitary as they age (Bercovitch et al. 2006; Carter et al. 2013; VanderWaal et al. 2014; Castles et al. 2019). Overall, these movement patterns give rise to fission-fusion dynamics in which individuals live in spatially overlapping communities whose group membership is constantly changing across short spatial and temporal scales (van der Jeugd and Prins, 2000; Bercovitch and Berry 2013; Carter et al. 2013; Bond et al. 2021a, 2021b). Recent studies have shown that giraffe social networks are flexibly structured, with associations influenced by ecological conditions, space use, and individual traits (Muller et al. 2019; Bond et al. 2021a, 2021b). Despite these advances, the behavioral mechanisms underpinning how groups coordinate movement within this fluid system remain poorly understood. While fission–fusion dynamics in giraffes have been well documented, there is limited empirical evidence linking individual characteristics and environmental context to collective movement decisions. In particular, it is unknown which individuals initiate group movements, how group members respond, and under what conditions groups remain cohesive or split. Understanding these processes is essential for bridging the gap between observed giraffe social structure and the behavioral mechanisms that generate it.

In this study, we investigated the mechanisms underlying collective movement decisions in a wild giraffe population. Our goal was to evaluate the individual, social, and ecological drivers of movement initiation and group cohesion. We tested 3 hypotheses (H1-3), drawing on collective behavior and fission-fusion literature across many species. (H1) Movement decisions are driven by adult females. In many matrilineal ungulate societies, adult females face high reproductive and nutritional demands (eg during lactation) and are often observed initiating and leading group movements to secure access to optimal foraging sites (Fischhoff et al. 2007; Berry and Bercovitch 2015). Within this context, we predicted that adult female giraffes would initiate group movements more frequently than individuals of other age-sex classes. Given the social dependence of younger group members on their mothers, we also predicted that subadults would follow movements initiated by adult females more quickly than individuals in other age-sex categories. Thus, movements initiated by adult females should be more likely to result in successful group relocation than movements initiated by other age-sex groups. (H2) Environmental context has a strong influence on movement decisions. Animals are more likely to constantly move within habitats where food resources are more scattered than concentrated. Additionally, more open habitats facilitate visual contact, thereby reducing coordination costs. Thus, we predicted that giraffes are more likely to initiate group movements within more open than closed habitats. (H3) Group cohesion during movements is constrained by coordination costs. Individual interests and preferences become more divergent as group size increases, making coordinated movement more difficult. Therefore, we predicted that, given movement initiation, group fission was more likely in larger groups.

## Materials and methods

### Data collection

We conducted fieldwork in the northern section of Murchison Falls National Park (MFNP; Fig. 1), in northwestern Uganda. MFNP is bisected by the River Nile into the northern and southern sections. The northern section supports a naturally occurring giraffe population of nearly 1,800 individuals (Kasozi et al. 2023; Brown et al. 2025). Recent conservation efforts led to the translocation of a small number of individuals to the southern section (Brown et al. 2025). The large population in the northern section provided a broad pool of individuals from which we conducted our collective movement observations, reducing the likelihood that the observed patterns were driven by only a few repeatedly encountered individuals. Between January and March 2024, we observed giraffe groups across 2 daily sessions, corresponding to peak giraffe activity during the day (ie morning, 8 AM to 1 PM, and afternoon, 3 PM to 6 PM) (Kasozi et al. 2022). We drove along the existing dirt road network, traversing different habitats to search for giraffe groups. To minimize observation bias, we randomized search routes across days and observed each group along the routes once a day. Given the cognitive and coordination constraints in collective movements, we adopted a conservative definition of group membership. There is wide variation in the nearest-neighbor distances used in previous giraffe group definitions (ie from 100 m (Prehn et al. 2019) to 1,000 m (Muller 2018)). Given that we conducted our observations across different habitat types (ie open grasslands, open woodlands, and closed woodlands), it was not possible to observe individuals beyond 200 meters in all habitat contexts. As such, we defined a group as 2 or more individuals within 200 meters of each other that were not moving in divergent directions at the onset of observation. We adopted this definition to capture socially and behaviorally cohesive units in which information sharing and coordinated movement decisions were most likely to occur.

**Figure 1:**
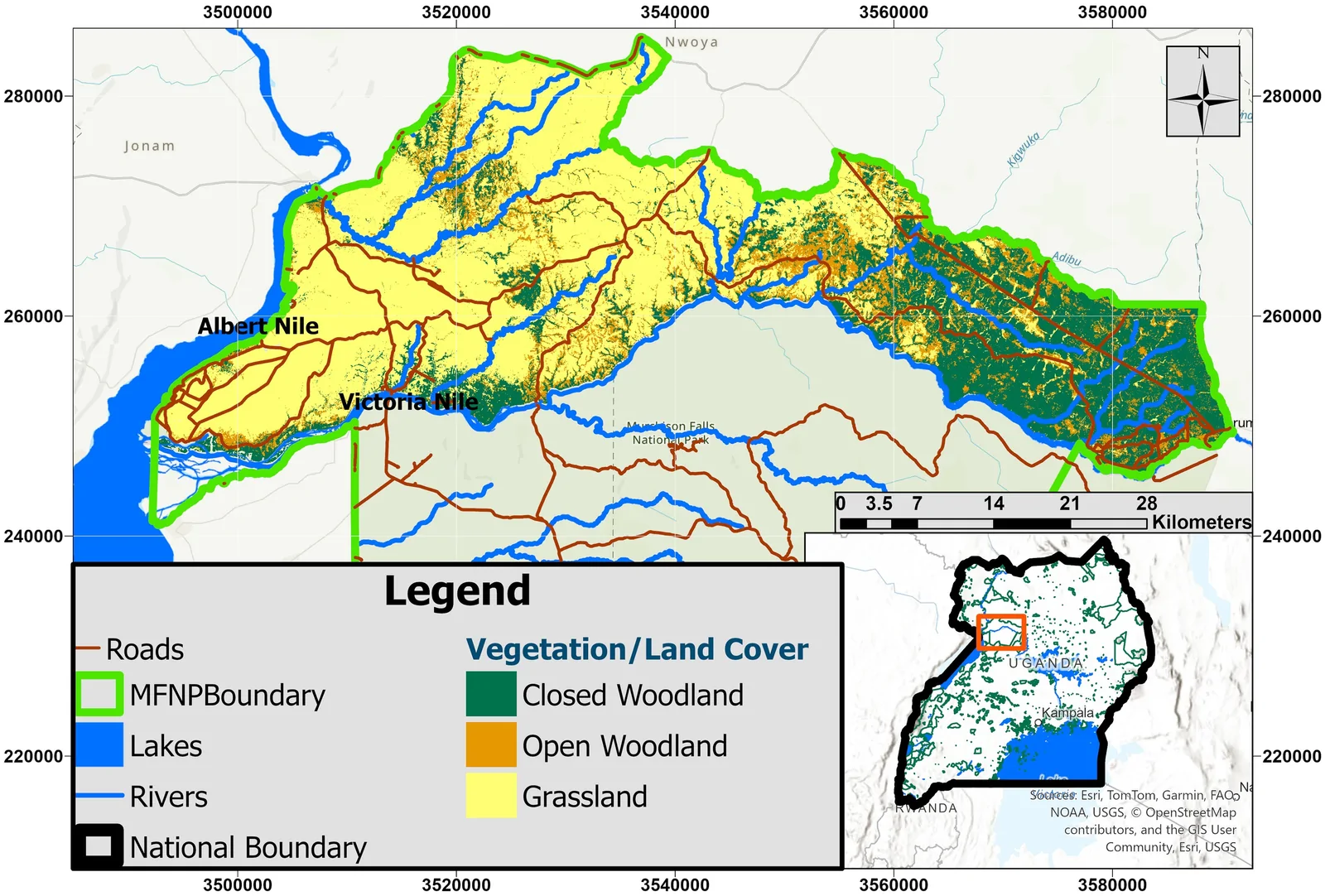
Location of MFNP, in northwestern Uganda, where giraffe collective movement observations were conducted.

We directly observed giraffe behavior using scan sampling (Altmann 1974) and scored all data using CyberTracker 3.0 software (CyberTracker Conservation, Bellville, SA; http://www.cybertracker.org/) installed on a Samsung Galaxy Tab S7+ mobile device. We observed each giraffe group we encountered on any given day for 60 min to monitor group dynamics. The fact that the giraffe population in MFNP comprises nearly 1,800 free-ranging individuals provided us with the opportunity to observe different groups on the same day (Kasozi et al. 2023; Brown et al. 2025). Given that we did not individually identify group members during the observations, we did not track groups longitudinally during sampling. Thus, we defined each group at the point of observation. Moreover, we considered each group observation across sampling days as unique and independent, given that giraffe group membership changes constantly across space and time (Carter et al. 2013; Muller et al. 2018; Bond et al. 2021a, 2021b). Before movement initiation within each group, we recorded the instantaneous activity of each group member at 10-min intervals. We categorized the activities as inactive (ie resting, ruminating, vigilant/scan) and active (ie feeding, walking, necking), and recorded the proportion of individuals engaged in each before movement initiation. We defined the observed activities following Seeber et al., (2012) as; resting (individuals lying down with head held high or placed on the body), ruminating (chewing already ingested food after regurgitation), vigilant/scan (an individual standing still with erect neck watching its surrounding), feeding (ingesting browse), walking (locomotion without engaging in feeding), and necking (2 individuals swinging head and neck against each other's body).

If no movement was observed after 60 min, data scoring was terminated, and we searched for another group. Conversely, when movement was initiated, we stopped recording the instantaneous behaviors of group members to focus on recording movement dynamics. We defined movement initiation as the spatial displacement of 1 or more individuals away from the predeparture area and other group members in a constant direction, without changing course, for at least 100 meters (Petit et al. 2009; Ramos et al. 2015). We used the 100-meter threshold to distinguish collective movement initiation from fine-scale repositioning within groups. Followers could begin responding before the initiator completed the 100-meter displacement, because collective movement decisions often emerge progressively through social interactions and coordinated responses among group members before complete spatial separation or group displacement occurs (Pillot and Deneubourg 2010). The initiator's spatial displacement was estimated from its departure position to the location when it reached the 100 m threshold used to define an initiation event. All distance measurements were taken from a stationary vehicle using a handheld laser rangefinder. We recorded the sex (male and female) and age (calf [0 to 12 mo], sub-adult [female: 1 to 3 yr, male: 1 to 6 yr], and adult [female: > 3 yr, male: > 6 yr]) of the movement initiator (Strauss et al. 2015). We used physical characteristics (ie body size, height, and ossicone development) to classify individuals into age classes. Body size and height increase progressively from calves to adults. Younger individuals have fringed hair at the tips of their ossicones, which wears off in adulthood. Adult males exhibit more pronounced ossicone development (including a prominent third ossicone) compared with subadult males. Additionally, we recorded the group size, group type (ie female-only, male-only, and mixed), and habitat type (ie open woodland, open grassland, and closed woodland) in which the group was observed. We broadly categorized the habitats based on vegetation composition and canopy cover, following structural distinctions commonly adopted in African savannah ecosystems (Scholes and Archer 1997; Bond 2008). Within this context, we defined grassland as areas dominated by herbaceous vegetation with sparse woody cover; open woodland as grass matrix with scattered woody vegetation and a discontinuous canopy; and closed woodland as areas dominated by woody vegetation with a near-continuous canopy (Scholes and Archer 1997; Bond 2008; Abdi et al. 2022).

We considered a movement initiation successful if at least 1 group member in the predeparture area followed the initiator. A follower was defined as any individual who moved in the same direction as the initiator within 15 min for at least 50 meters (Ramos et al. 2015). If no group member followed the initiator within 15 min, we regarded the initiation attempt as unsuccessful. Limiting follower-latency observations to 15 min assumes individuals respond to the initiator's cue, as coordinated decisions typically occur within short time frames. Movements beyond 15 min are likely driven by independent motivation rather than response to the initiator. For each follower, we recorded the latency (in seconds) to follow the initiator (hereafter “follow latency”), along with their age class (defined above) and sex. Any individual leaving the predeparture area at an angle >45° to the left or right of the initiator's direction was considered an initiator of a separate departure attempt (Sueur and Petit 2008; Pyritz et al. 2011). We estimated the angle visually using the inbuilt compass of a handheld GPS device. Consequently, we defined fission events as those with 2 initiations in different directions or as those in which at least 2 group members moved while others remained in place. We did not consider unsuccessful movement initiations as fission events. After observing a group collective movement event, we searched for other groups to maximize spatial coverage and avoid repeated sampling of the same individuals. All observations were made from a stationary vehicle, with the engine switched off, at a minimum distance of at least 100 meters from the animals to avoid disturbing their natural behaviors. Giraffes within MFNP appear to be habituated to humans in a vehicle, perhaps due to long-term exposure to tourism, management, and oil and gas operations (Pomeroy et al., 2024). Giraffe tolerance to vehicles has been reported elsewhere (Bond et al. 2021a).

### Statistical analysis

We combined descriptive statistics with logistic regression and survival modeling to assess the characteristics of initiators of successful group movements and followers, the social and ecological factors associated with the initiation of group movements, the group-level outcomes of movement initiations, and the latency to follow.

#### Initiator and follower propensities

Since individuals across age-sex categories were unequally represented within groups during sampling, we calculated initiator and follower propensities by standardizing behavioral events relative to the demographic distribution in our sample. We therefore expressed initiation and follower propensities as the respective proportions of initiation or follower events performed by individuals of each age-sex category, divided by the proportional representation of that category during the sampling period. This normalization approach is crucial in minimizing bias caused by demographic overrepresentation within social groups. As a result, the propensities reflect behavioral tendencies rather than differences in demographic representation within social groups.

#### Predictors of successful movement initiations

To examine the ecological and social factors associated with the likelihood of initiating a successful group movement (as defined above), we used a generalized linear mixed model (GLMM) with a binomial error distribution and a logit link function. We coded the response variable (ie Movement) as “1” to represent a movement initiation in which at least 1 group member followed, and “0” otherwise. We included date of observation as a random effect in the model to account for potential nonindependence in observations that could arise from shared environmental conditions among groups sampled on the same days and to facilitate generalization beyond our observation days (Gomes 2022).

We specified fixed effects as: habitat type (closed woodland, open grassland, and closed bushland as the reference); group type (male only, female only, and mixed-sex as the reference), time period (Evening and Morning as the reference), group size and proportion active (ie the proportion of group members engaged in active behavior at the time of initiation). We fitted the model using the “glmer” function from the lme4 package (Bates et al. 2015) in R (version 4.2.2, R Core Team 2022). We standardized the continuous predictors (group size and proportion of active individuals) to a centered mean of 0 and a standard deviation of 1. Standardizing facilitates model convergence and direct comparison of effect sizes since the variables are measured on different scales. The statistical significance of the fixed effects was assessed by calculating Wald *z*-values and corresponding *P*-values at the 0.05 α-level, which are automatically provided by the model “summary” function in lme4. Model convergence and fit were assessed using the “check_model” and “check_collinearity” functions from the performance package (Lüdecke et al. 2021). We assessed multicollinearity among the predictor variables using Generalized Variation Inflation Factors (GVIFs), where scores of <5 represented low collinearity and scores >5 represented high collinearity. Model goodness of fit was assessed using log-likelihood values and inspection of residual plots (Sutherland et al. 2023). The predictive performance of the model was evaluated using the area under (AUC) the Receiver Operating Characteristic (ROC) curve (Pontius and Parmentier 2014). The ROC curve assesses the model's ability to discriminate between false and true positive outcomes across a range of threshold values (Pontius and Parmentier 2014). AUC quantifies the model's discriminatory ability, with values < 0.5 indicating low ability and values > 0.5 indicating high ability (Pontius and Parmentier 2014).

#### Predictors of movement outcomes

We identified 2 outcomes of movement initiations within groups: fission (partial group splitting) and cohesion (where all group members move). We therefore modeled the movement outcome as a binary response, coded as 1 for fission and 0 for no fission. We included social factors (group size, proportion of active individuals, and group type), environmental factors (habitat type), and observation (time period) as predictors. We standardized the continuous predictors (group size and proportion of active individuals) to a centered mean of 0 and a standard deviation of 1. Due to unequal representation of fission and cohesion events and a relatively small sample size (data subset including only movement initiation events), we fitted Firth's bias-reduced penalized-likelihood logistic regression (Firth 1993). The bias-reduced model addresses potential bias resulting from small sample sizes and quasi-complete separation in the fission-cohesion dataset. The approach used by the model provides more stable coefficient estimates and reliable inference when response categories are unevenly represented (Firth 1993; Mansournia et al. 2018). We fitted the model using the “brglm” function from the brglm2 package in R (Kosmidis 2025).

#### Predictors of follower latency

To examine the factors influencing the follower latencies, we fitted a clustered Accelerated Failure Time (AFT) model to follower data. We fitted this model using the “survreg” function from the survival package (Therneau 2024) in R (R Core Team 2022). We assumed that the baseline hazard function h_0_ (t) comes from the Weibull distribution with shape parameter *a* > 0 and scale parameter *b* > 0. We then defined the baseline hazard function as h_o_(*t*) = (*at^a^*^−1^)/*b^a^* (Król et al. 2017). We defined the response variable as follow latency, corresponding to the time elapsed (in seconds) from movement initiation to the ith following event. We right-censored the following time at 900 seconds and created a binary response variable (ie Status), with a value of 1 for events occurring within 900 seconds and 0 otherwise. We included follower sex, initiator sex, follower age, group type, and group size as fixed effects. To account for the nonindependence of follower events within the social groups, we computed clustered standard errors using “Group_ID” as the clustering variable. We initially treated “Group_ID” as a shared frailty term to account for nonindependence of follower events within groups, but the model estimated very low frailty variance (θ = 0.16).

All statistical analyses were conducted using RStudio version 2026.04.0+526 (Posit team 2026), running R version 4.2.2 (R Core Team 2022), with a significance level set at 0.05.

## Results

We observed 169 giraffe groups ranging in size from 2 to 31 individuals (mean = 15, SD = 7.4, Figure S1) across 260 h. The majority of groups were observed in the morning (63.9%, *n* = 108), while the rest were observed in the evening (36.1%, *n* = 61). Furthermore, the majority of the observed groups consisted of both males and females (84.6%, *n* = 143), while the remaining groups were either female-only (7.7%, *n* = 13) or male-only (7.7%, *n* = 13). Across the sampling period, adult females constituted the largest proportion of individuals recorded (48.4%, *n* = 898), followed by adult males (34.4%, *n* = 637), subadult males (7.1%, *n* = 131), and subadult females (6.9%, *n* = 128). Calves accounted for only 3.2% of all giraffes observed (*n* = 60).

### Initiator characteristics

We recorded movement in 118 groups and no movement in 51 groups. Only 1 initiation attempt was unsuccessful. Furthermore, we recorded only 1 instance of 2 simultaneous initiations within the same group. Most movement initiations (*n* = 84) were performed by adult females, resulting in an initiation propensity of 9.4% relative to their availability in the sample. Conversely, males initiated 35 group movements, with an initiation propensity of 5.5%. We did not observe movement initiations from subadults or calves of either sex.

### Group movement outcomes

Among the 118 group movements, the majority resulted in a fission event (*n* = 79, 66.9%), while the rest (*n* = 39, 33.1%) led to cohesion. Group fission was observed more in the evening (*N* = 43 groups, number of fission events, *n* = 35, 81.4%) than in the morning (*N* = 75 groups; number of fission events, *n* = 44, 58.7%).

### Follower characteristics

We recorded 636 follower events, distributed among adult females (*n* = 288, 45.3%), adult males (*n* = 215, 33.8%), subadult males (*n* = 56, 8.8%), subadult females (*n* = 55, 8.6%), and calves (*n* = 22, 3.5%). However, relative to their availability in the sample, subadult females had the highest following propensity (*n* = 103; follow propensity = 53.4%), followed by subadult males (*n* = 108; follow propensity = 51.9%), calves (*n* = 44; follow propensity = 50%), adult males (*n* = 464; follow propensity = 46.3%), and lastly adult females (*n* = 648; follow propensity = 44.4%).

### Predictors of movement initiations

Movement initiation probability increased significantly with group size (*β* = 0.37 ± 0.10, *P* < 0.001, Table 1) and the proportion of active individuals within the group (*β* = 1.21 ± 0.09, *P* < 0.001, Fig. 2a). Group movement initiations were more likely to occur in the evening (*β* = 0.78 ± 0.16, *P* < 0.001). Female-only groups exhibited lower movement initiation probability than mixed groups (*β* = −1.22 ± 0.39, *P* = 0.0016; Fig. 2b). Habitat type also influenced the likelihood of group movement initiation, with groups in closed woodland being less likely to move than those in open habitats (*β* = −1.93 ± 0.28, *P* < 0.001; Fig. 2c). The random intercept variance for date (*σ*^2^ = 2.77) indicated substantial daily variation in movement propensity.

**Figure 2:**
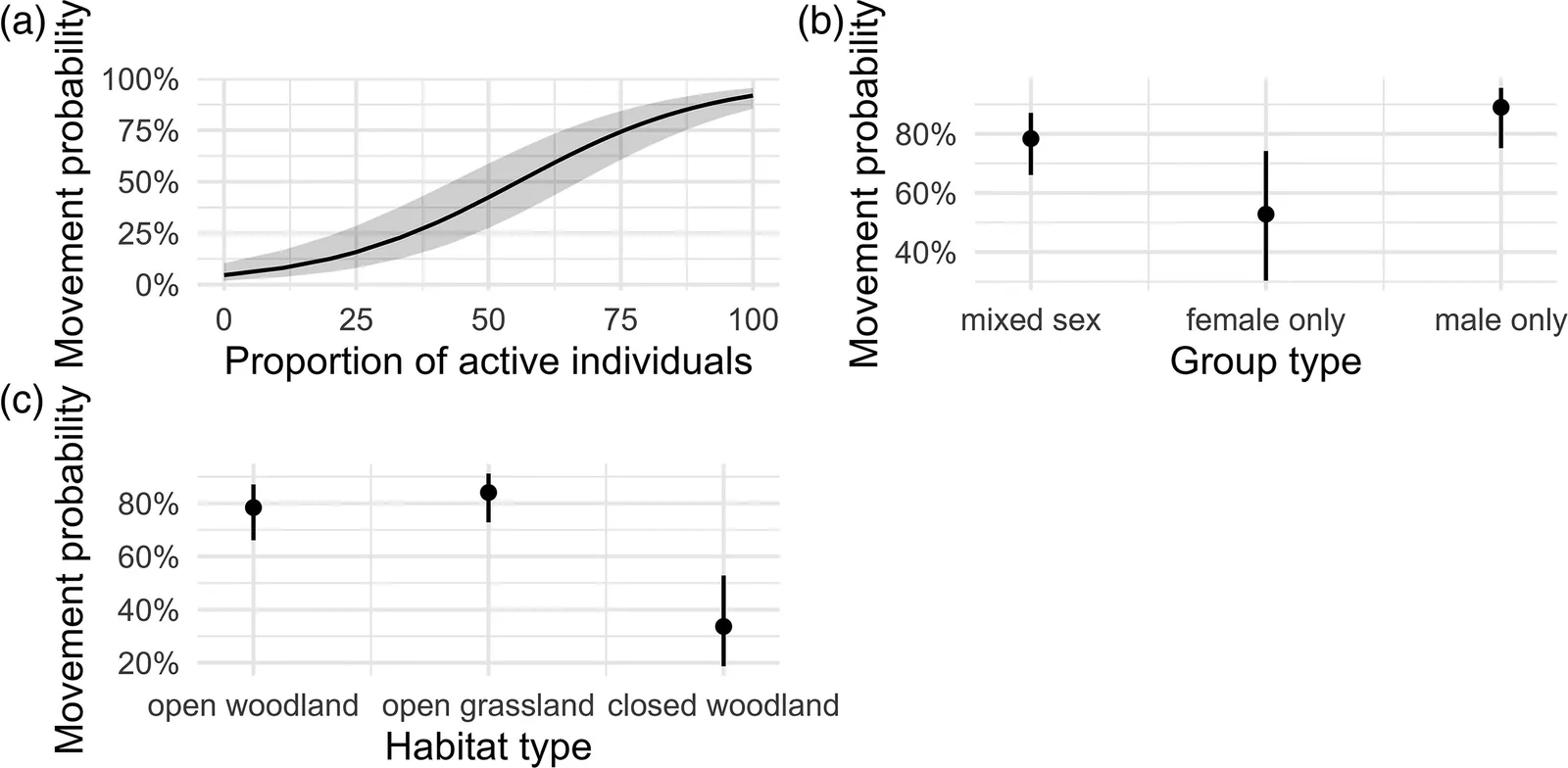
Predicted probability of group movement as a function of: a) proportion of active individuals in the group at the time of initiation, b) Group type, and c) Habitat type.

**Table 1:** Parameter estimates of the GLMM fitted to giraffe collective movements data.

	Movement		
Predictors	Log-Odds	CI	*P*
(Intercept)	1.29	0.67–1.91	<0.001
Habitat type [open grassland]	0.37	−0.02–0.77	0.065
Habitat type [closed woodland]	−1.93	−2.48 to −1.37	<0.001
Group type [female only]	−1.22	−1.98 to −0.46	0.002
Group type [male only]	0.68	−0.08–1.45	0.079
Time Period [evening]	0.78	0.47–1.10	<0.001
Group size	0.37	0.16–0.57	<0.001
Prop active	1.21	1.03–1.39	<0.001
**Random Effects**			
σ^2^	3.29		
τ_00 Date_	2.77		
ICC	0.46		
N _Date_	34		
Observations	1,853		
Marginal *R*^2^/Conditional *R*^2^	0.233/0.584		

The model converged with no indication of over dispersion (dispersion ratio = 0.9, *P* = 0.5). Additionally, all the predictor variables had adjusted generalized variation inflation factor values below 1.1 (Table S1). Finally, the model demonstrated a strong distinction between successful and nonmovement events, as indicated by an AUC of 0.78 (Figure S2).

### Predictors of movement outcomes

The bias-reduced logistic regression model identified several significant predictors of group fission (Table 2). The likelihood of fission increased markedly with group size (*β* = 4.37 ± 0.32, *z* = 13.49, *P* < 0.001, Fig. 3) and was substantially higher in male-only (*β* = 3.02 ± 0.57, *P* < 0.001) and female-only groups (*β* = 1.12 ± 0.40, *P* = 0.005) compared with mixed groups. The probability of fission was higher in open habitats (*β* = −2.34 ± 0.45, *P* < 0.001) relative to closed-woodland habitats. The proportion of active individuals before movement also negatively predicted fission occurrence (*β* = −0.38 ± 0.10, *P* < 0.001). There was no significant effect of time of day on fission probability (*β* = 0.29 ± 0.23, *P* = 0.20). The model explained a large portion of deviance (residual deviance = 685.3 vs. null deviance = 1,273.5), demonstrating strong explanatory power and overall good calibration (AUC ≈ 0.83, Figure S3). Additionally, all the predictor variables had adjusted generalized variation inflation factor values below 2.1 (Table S2).

**Figure 3:**
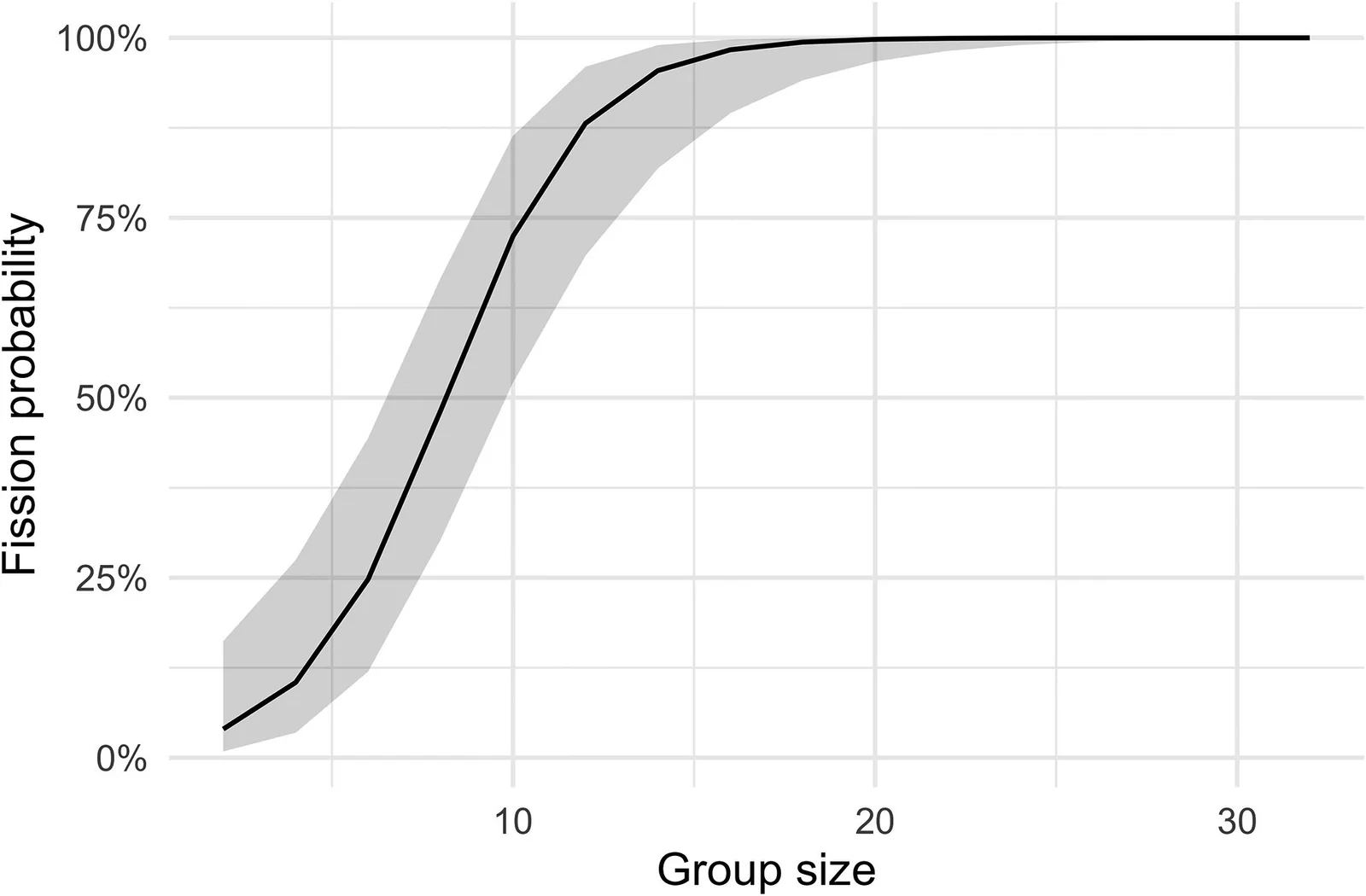
Predicted probability of group fission, given movement initiation, as a function of group size.

**Table 2:** Parameter estimates of the Penalized Likelihood Logistic Regression Model fitted to giraffe collective movements data.

	Movement outcome		
Predictors	Log-Odds	CI	*P*
(Intercept)	4.16	3.55–4.91	<0.001
Habitat type [open grassland]	0.07	−0.36–0.51	0.734
Habitat type [closed woodland]	−2.34	−3.26 to −1.46	<0.001
Group type [female only]	1.12	0.33–1.93	0.005
Group type [male only]	3.02	1.96–4.27	<0.001
Time period [evening]	0.29	−0.15–0.74	0.202
Group size	4.37	3.76–5.11	<0.001
Prop active	−0.38	−0.59 to −0.19	<0.001
Observations	1,367		
*R* ^2^ Tjur	0.475		

### Predictors of follower latency

The Weibull AFT model revealed significant effects of group size on follower latency (Table 3). Subadult followers exhibited significantly shorter latencies compared with adults (*β* = −0.868, *P* = 0.0007, Fig. 4a). Individuals within larger groups exhibited delayed responses to movement initiations when compared with smaller groups (*β* = 0.179, *P* < 0.001). Neither follower sex (Fig. 4b) nor initiator sex significantly affected latency, and differences among group types (female-only, male-only, mixed-sex) were weak and nonsignificant. The shape parameter of the Weibull distribution was less than 1 (shape = 0.62), suggesting that the hazard of following decreases over time (ie the longer a follower waited, the less likely they were to join).

**Figure 4:**
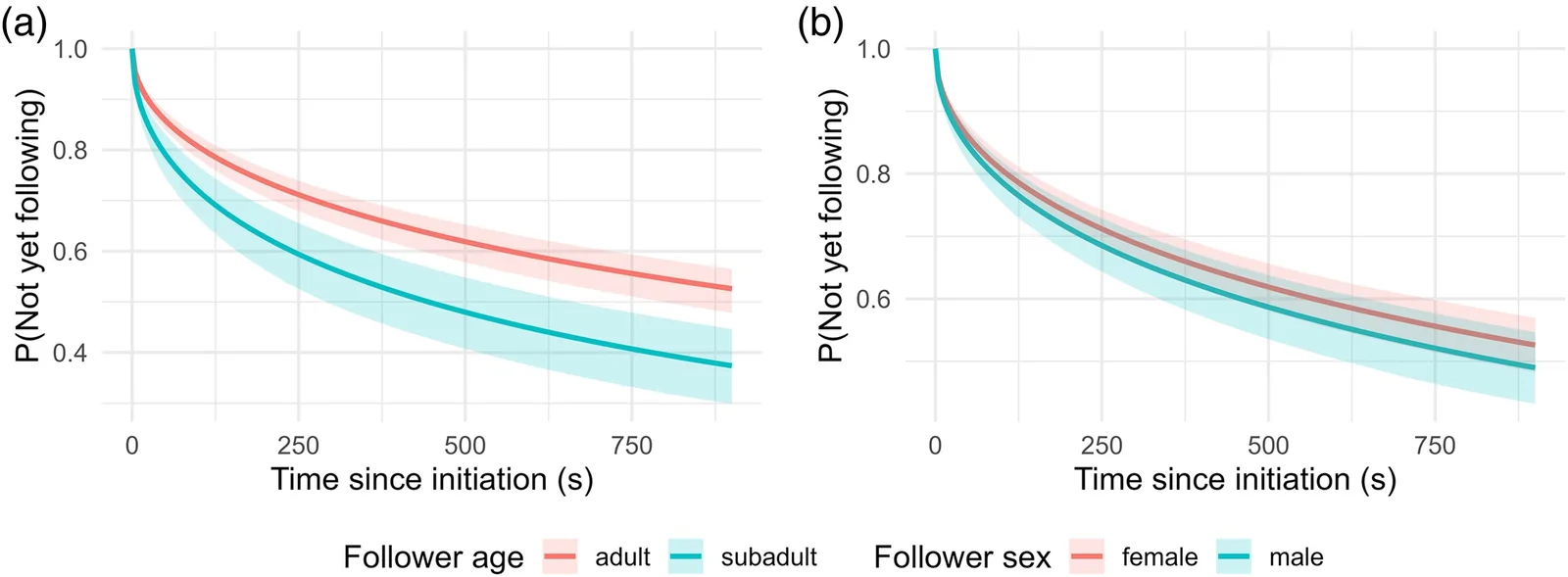
Weibull AFT curves showing differences in follower latency across age–sex categories. a) Subadult individuals exhibited shorter follower latencies than adults, as indicated by a more rapid decline in the probability of not yet following over time. b) Males followed slightly faster than females. Shaded bands represent 95% confidence intervals.

**Table 3:** Parameter estimates of the robust AFT model fitted to giraffe collective movements data.

	Value	Std. Err	(Naive SE)	*z*	*P*
(Intercept)	5.70	0.48	0.38	11.79	<0.000
Follower sex [male]	−0.21	0.21	0.18	−1.00	0.315
Initiator sex [male]	0.13	0.31	0.20	0.41	0.682
Follower age [subadult]	−0.86	0.26	0.22	−3.36	0.001
Group type [male only]	0.07	0.78	0.57	0.09	0.925
Group type [mixed sex]	−0.50	0.50	0.40	−0.99	0.324
Group size	0.18	0.02	0.01	11.27	< 0.000
Log(scale)	0.70	0.03	0.04	20.48	< 0.000

## Discussion

Overall, the results largely supported our a priori predictions about movement initiation, follower responses, environmental context, and collective movement outcomes. Specifically, adult females initiated a higher proportion of successful collective movements, movement initiation was more prevalent in open habitats, larger groups were more likely to split upon movement, and subadults followed initiators more rapidly than adults. Together, these findings suggest that giraffe collective movements are driven by a structured decision-making process shaped by individual, social, and ecological conditions.

We hypothesized that adult females are influential drivers of movement decisions during giraffe group movements. Consistent with this hypothesis, we found that adult females exhibited a higher propensity to initiate group movements than adult males. Female-biased leadership has been observed across a range of other large mammals (Bourjade and Sueur 2010; Mutinda et al. 2011; Briard et al. 2015; Ramos et al. 2015). For example, among plains zebras (*Equus quagga*), lactating females have been observed to lead harems with higher frequencies (Fischhoff et al. 2007). Similar trends have been observed among elephants, where matriarchs play a pivotal role in leading herds (McComb et al. 2011). Lactating females perhaps search for high-return forage and safe habitats for their young. Additionally, as they age, adult females gain valuable experience of the habitat, from which other group members can draw. Individual knowledge and experience have been recorded as key determinants of feeding-site selection among large mammals such as American bison (*Bison bison*) (Merkle et al. 2014). Overall, variation in physiological requirements (including protein, energy, and water needs) across sex and age groups likely drives individual propensity to move (Sueur et al. 2009). As such, movement could be initiated without needing dominance, complex signals, or advanced cognitive abilities (Sueur et al., 2011).

Although this occurred to a lesser degree than in females, our results highlight a substantial contribution of adult males initiating successful group movements. Adult male giraffes exhibit loose associations characterized by constant roaming movements among female groups to seek mating opportunities (Bercovitch et al. 2006; Carter et al. 2013; Ferres et al. 2021). Within MFNP, recent work highlighted long distance partial migration of some adult males between the eastern and western sections of the landscape (Brown and Bolger 2020). While adult male movement strategies may influence fewer collective movements, they contribute substantially to maintaining social connectivity (Ferres et al. 2021), and could potentially facilitate genetic flow within giraffe populations (Lohay et al. 2023). Overall, variation in physiological needs, reproductive strategy, and age class likely influence the propensity to initiate movements within giraffe groups.

Individuals were more likely to follow movement initiations when a higher proportion of group members were in active behavioral states, such as feeding, walking, or necking, at the time of initiation. Active behavior can create positive feedback loops that encourage coordinated behavior within the group (Conradt and Roper 2005). The heightened alertness and engagement of individuals in active behavioral states increase their responsiveness to movement initiation cues, leading to better alignment and synchronization among group members (Conradt and Roper 2005). Among social ungulates, actions of a few individuals can quickly spread through the group to initiate collective behavior (King et al., 2009). For example, among sheep and cattle (*Ovis aries*), spontaneous group movements are significantly influenced by the social interactions and activity levels of individuals (Ramseyer et al. 2009). Similar observations have been made in nonungulates such as Canada geese (*Branta canadensis*) (Raveling 1969), and African elephants (Moss 1988). Overall, active behavior among group members prior to departure can facilitate response to the movement initiation cue, alignment, and hence successful collective movement (Couzin et al. 2005; Rieucau and Giraldeau 2011).

We found a significant positive relationship between group size and the probability of initiating a group movement. Similarly, larger group sizes were associated with slower response times (ie longer latencies) to movement initiation, supporting our hypothesis that group cohesion during movements is constrained by coordination costs. Increasing group size perhaps increases resource competition, and prompts individuals to move. Furthermore, a large group size potentially constrains collective movement due to increased coordination demands (Sundaresan et al. 2007). Signals and cues communicating intent to move may take longer to be disseminated throughout larger groups (Liao et al. 2024). Additionally, consistent with our expectations, we observed that the successful initiation of group movements was more likely in open grassland habitats than in closed woodlands. Within closed woodland habitats, food resources for giraffes occur in higher density, thus diminishing the need for constant movement. Additionally, within wooded habitats, group members may need to stay close to each other to reduce predation risk due to decreased predator visibility. By contrast, within open grassland habitats, giraffes need to move constantly to find woody vegetation that comprises a bulk of their diet. Additionally, in such habitats, giraffes are likely less averse to predation risk, given their heightened ability to detect any predators. Moreover, movement cues are likely to spread more easily throughout groups in open habitats, where individuals can see each other more easily.

Group fission in giraffes was strongly linked to group size, composition, and habitat structure. Larger groups were more likely to split upon movement (Fig. 3), supporting the hypothesis that coordination costs, which increase with group size, constrain group cohesion during group movement. The high tendency for male-only groups to split aligns with known patterns of weaker social bonds among males and frequent turnover in bachelor groups, consistent with their roaming strategy of seeking for mates (Ferres et al. 2021). In contrast, female-only groups, though more cohesive, also tended to divide, possibly due to reproductive or maternal differences in the timing of activity.

As predicted, we found that environmental context strongly influences giraffe group movements. In particular, the significant negative impact of closed-woodland habitats on fission indicates that visual isolation and limited movement space may strengthen the benefits of group cohesion. Given that dense woody vegetation diminishes visual contact among group members, they may be constrained to stay close to one another to increase collective awareness of their environment and minimize the risk of predation. In contrast, open grasslands provide better visibility, reducing the predation-related advantages of maintaining very close contact. Given that ecological pressures promoting group cohesion are relaxed in these habitats, individuals could move more readily, resulting in subgroup formation, as they can maintain visual contact and environmental awareness over greater distances. These results highlight how ecological context and group structure shape fission–fusion dynamics in giraffes. Similar patterns have been documented in other societies with high degrees of fission–fusion dynamics, where flexible subgroup formation helps animals balance the costs and benefits of sociality under changing ecological and social conditions (Markham and Gesquiere, 2017; Wielgus et al. 2020; Madsen and de Silva 2024). Since habitat openness and group size strongly influence splitting behavior, the ongoing landscape changes in MFNP that alter visibility and resource distribution (eg habitat clearance and fragmentation from infrastructure developments linked to oil and gas extraction) may significantly affect giraffe social organization and the frequency of fission events. Recent work has documented weaker relationship strengths and more exclusive associations among giraffes inhabiting human-dominated habitats (Bond et al. 2021a). Therefore, anthropogenic changes that alter habitat structure may disrupt information transfer, social cohesion, and movement connectivity within animal populations (Banks et al. 2007).

As highlighted by our AFT analysis, several factors influence follower latencies. Consistent with our predictions, we found that subadult individuals responded more quickly to movement cues than other age groups, which is likely due to their reliance on older group members for safety and resource acquisition (Ramos et al. 2015), and the influence of kin-based associations within giraffe groups (Bercovitch and Berry 2013). Because female giraffes are generally philopatric and maintain long-term associations with offspring and related individuals (Bercovitch and Berry 2013; Ferres et al. 2021), subadults may be more likely to rapidly follow familiar or maternally associated group members during collective movements. Therefore, the variation in response times among group members to movement stimuli might reflect age-related differences in social motivation, learning, and risk perception. Additionally, the slower response times observed in larger groups may stem from higher coordination costs and conflicting needs among members. Our model results showed a decreasing hazard in the Weibull distribution, indicating a temporal threshold in individual joining times. Consequently, the likelihood of joining a movement initiation decreased as response times increased. Overall, these findings suggest that both social composition and group size influence how quickly giraffes coordinate their movements, with smaller, adult-led groups achieving faster cohesion. Unmeasured group-level factors, such as prior experience of members and social structure, may also impact decisions to follow, and we recommend that future studies incorporate these factors.

While our study revealed broad patterns in collective movement decisions among giraffes, we highlight key limitations and future directions. Although our sample size was sufficient to detect general movement patterns associated with individual, social, and ecological factors, longer-term studies would enable more detailed analyses of finer-scale variation in giraffe collective movement dynamics. Additionally, we did not individually identify giraffes or longitudinally track specific individuals or groups, limiting our ability to assess whether consistent leadership by specific individuals shaped collective decisions. Thus, we have highlighted the broad roles of individuals across different age-sex classes in giraffe collective movements. Future work incorporating individual identification, social network analysis, and continuous tracking of individuals or groups would yield additional insights into leadership consistency, the duration of group cohesion and timing of fission events, and the temporal drivers of collective movement and subgrouping.

In conclusion, we have examined the multidimensional decision-making processes underlying giraffe collective movements and their associated group-level outcomes. Individual characteristics, such as age and sex, along with social factors like group size and type, as well as environmental influences, including habitat type, all play crucial roles in shaping giraffe group movements and fission dynamics. Our results provide a quantitative framework that links individual decisions to group-level outcomes in a large mammal species with a high degree of fission-fusion dynamics. We emphasize that fission dynamics emerge from the underlying framework of coordination processes, upon which collective movements are based. Group fission events are not random but predictable consequences of group composition and individual responses to movement initiation. We hope this study inspires further exploration of decision-making, coordination, and information exchange in giraffes and other large social mammals that live in societies with a high degree of fission-fusion dynamics.

## Supplementary Material

arag098_Supplementary_Data

## Data Availability

Analyses reported in this article can be reproduced using the data provided by Kasozi et al. (2026). Data to reproduce the analysis in this manuscript is available at https://doi.org/10.6084/m9.figshare.30731690.

## References

[arag098-B1] Abdi AM, Brandt M, Abel C, Fensholt R. 2022. Satellite remote sensing of savannas: current Status and emerging opportunities. J Remote Sens. 2022:9835284. 10.34133/2022/9835284.

[arag098-B2] Altmann J . 1974. Observational study of behavior: sampling methods. Behaviour. 49:227–267. 10.1163/156853974X00534. 4597405

[arag098-B3] Aureli F et al 2008. Fission-fusion dynamics. Curr Anthropol. 49:627–654. 10.1086/586708.

[arag098-B4] Banks SC, Piggott MP, Stow AJ, Taylor AC. 2007. Sex and sociality in a disconnected world: a review of the impacts of habitat fragmentation on animal social interactions. Can J Zool. 85:1065–1079. 10.1139/Z07-094.

[arag098-B5] Bashaw MJ, Bloomsmith MA, Maple TL, Bercovitch FB. 2007. The structure of social relationships among captive female giraffe (Giraffa camelopardalis). J Comp Psychol. 121:46–53. 10.1037/0735-7036.121.1.46. 17324074

[arag098-B6] Bates D, Maechler M, Bolker B, Walker S. 2015. Fitting linear mixed-effects models Usinglme4. J Stat Softw. 67:1–48. 10.18637/jss.v067.i01.

[arag098-B7] Bercovitch FB, Bashaw MJ, del Castillo SM. 2006. Sociosexual behavior, male mating tactics, and the reproductive cycle of giraffe Giraffa camelopardalis. Horm Behav. 50:314–321. 10.1016/j.yhbeh.2006.04.004. 16765955

[arag098-B8] Bercovitch FB, Berry PSM. 2013. Herd composition, kinship and fission-fusion social dynamics among wild giraffe. Afr J Ecol. 51:206–216. 10.1111/aje.12024.

[arag098-B9] Berry PSM, Bercovitch FB. 2015. Leadership of herd progressions in the Thornicroft's giraffe of Zambia. Afr J Ecol. 53:175–182. 10.1111/aje.12173.

[arag098-B10] Bond ML, König B, Lee DE, Ozgul A, Farine DR. 2021a. Proximity to humans affects local social structure in a giraffe metapopulation. J Anim Ecol. 90:212–221. 10.1111/1365-2656.13247. 32515083

[arag098-B11] Bond ML, Lee DE, Ozgul A, Farine DR, König B. 2021b. Leaving by staying: social dispersal in giraffes. J Anim Ecol. 90:2755–2766. 10.1111/1365-2656.13582.

[arag098-B12] Bond WJ . 2008. What limits trees in C4 grasslands and savannas? Ann Rev Ecol Evol Syst. 39:641–659. 10.1146/annurev.ecolsys.39.110707.173411.

[arag098-B13] Bourjade M, Sueur C. 2010. Shared or unshared consensus for collective movement? Towards methodological concerns. Behav Processes. 84:648–652. 10.1016/j.beproc.2010.02.027. 20211230

[arag098-B14] Briard L, Dorn C, Petit O. 2015. Personality and affinities play a key role in the organisation of collective movements in a group of domestic horses. Ethology. 121:888–902. 10.1111/eth.12402.

[arag098-B15] Brown MB et al 2025. Assessing the success of conservation translocation establishment: post-translocation demography of Nubian giraffe (Giraffa camelopardalis camelopardalis) in Uganda. Conserv Sci Pract. 7:e70127. 10.1111/csp2.70127.

[arag098-B16] Brown MB, Bolger DT. 2020. Male-biased partial migration in a giraffe population. Front Ecol Evol. 7:524. 10.3389/fevo.2019.00524.

[arag098-B17] Carter KD, Seddon JM, Frère CH, Carter JK, Goldizen AW. 2013. Fission–fusion dynamics in wild giraffes may be driven by kinship, spatial overlap and individual social preferences. Anim Behav. 85:385–394. 10.1016/j.anbehav.2012.11.011.

[arag098-B18] Castles MP et al 2019. Relationships between male giraffes’ colour, age and sociability. Anim Behav. 157:13–25. 10.1016/j.anbehav.2019.08.003.

[arag098-B19] Conradt L, Roper TJ. 2005. Consensus decision-making in animals. Trends Ecol Evol. 20:449–456. 10.1016/j.tree.2005.05.008. 16701416

[arag098-B20] Conradt L, Roper TJ. 2009. Conflicts of interest and the evolution of decision sharing. Philos Trans R Soc Lond B Biol Sci. 364:807–819. 10.1098/rstb.2008.0257. PMC2689720 19073479

[arag098-B21] Couzin ID, Krause J, Franks NR, Levin SA. 2005. Effective leadership and decision-making in animal groups on the move. Nature. 433:513–516. 10.1038/nature03236. 15690039

[arag098-B22] Dagg A, Foster J. 1976. The giraffe: its biology, behavior, and reproduction. R.E. Krieger Publishing Company.

[arag098-B23] Duboscq J, Romano V, MacIntosh A, Sueur C. 2016. Social information transmission in animals: lessons from studies of diffusion. Front Psychol. 7:1147. 10.3389/fpsyg.2016.01147. PMC4973104 27540368

[arag098-B24] Ferres JML et al 2021. Social connectedness and movements among communities of giraffes vary by sex and age class. Anim Behav. 180:315–328. 10.1016/j.anbehav.2021.08.008.

[arag098-B25] Firth D . 1993. Bias reduction of maximum likelihood estimates. Biometrika. 80:27–38. 10.1093/biomet/80.1.27.

[arag098-B26] Fischhoff IR et al 2007. Social relationships and reproductive state influence leadership roles in movements of plains zebra (Equus burchellii). Anim Behav. 73:825–831. 10.1016/j.anbehav.2006.10.012.

[arag098-B27] Franks NR, Pratt SC, Mallon EB, Britton NF, Sumpter DJT. 2002. Information flow, opinion polling and collective intelligence in house-hunting social insects. Philos Trans R Soc Lond B Biol Sci. 357:1567–1583. 10.1098/rstb.2002.1066. PMC1693068 12495514

[arag098-B28] Gomes DGE . 2022. Should I use fixed effects or random effects when I have fewer than five levels of a grouping factor in a mixed-effects model? PeerJ. 10:e12794. 10.7717/peerj.12794. PMC8784019 35116198

[arag098-B29] Kasozi H et al 2026. Data from: The socioecological correlates of collective movements in giraffes. Behavioral Ecology. https://figshare.com/s/36435b32411498e60f48

[arag098-B30] Kasozi H, Linden DW, Roloff GJ, Montgomery R. 2023. Evaluating the prevalence and spatial distribution of giraffes injured by non-target poaching. J Zool. 319:152–162. 10.1111/jzo.13033.

[arag098-B31] Kasozi H, Moll RJ, Kityo RM, Montgomery RA. 2022. Phylogeny is a stronger predictor of activity than allometry in an African mammal community. Biol J Linn Soc Lond. 135:599–609. 10.1093/biolinnean/blab162.

[arag098-B32] Kasozi H, Montgomery RA. 2018. How do giraffes locate one another? A review of visual, auditory, and olfactory communication among giraffes. J Zool. 306:139–146. 10.1111/jzo.12604.

[arag098-B33] Kasozi H, Montgomery RA. 2020. Variability in the estimation of ungulate group sizes complicates ecological inference. Ecol Evol. 10:6881–6889. 10.1002/ece3.6463. PMC7391342 32760498

[arag098-B34] King AJ, Johnson DDP, Van Vugt M. 2009. The origins and evolution of leadership. Curr Biol. 19:R911–R916. 10.1016/j.cub.2009.07.027. 19825357

[arag098-B35] King AJ, Sueur C. 2011. Where next? Group coordination and collective decision-making by primates. Folia Primatol (Basel). 32:1245–1267. 10.1007/s10764-011-9526-7.

[arag098-B36] Kosmidis I . 2025. brglm2: bias reduction in generalized linear modelsR package version 1.0.0 [R Package], https://CRAN.R-project.org/package=brglm2.

[arag098-B37] Krause J, Ruxton GD. 2002. Living in groups. Oxford University Press.

[arag098-B38] Król A et al 2017. Tutorial in joint modeling and prediction: a statistical software for correlated longitudinal outcomes, recurrent events and a terminal event. J Stat Softw. 81:1–52. 10.18637/jss.v081.i03.

[arag098-B39] Liao C-C, Magrath RD, Manser MB, Farine DR. 2024. The relative contribution of acoustic signals versus movement cues in group coordination and collective decision-making. Philos Trans R Soc Lond B Biol Sci. 379:20230184. 10.1098/rstb.2023.0184. PMC11391321 38768199

[arag098-B40] Lohay GG et al 2023. Genetic evidence of population subdivision among Masai giraffes separated by the Gregory Rift Valley in Tanzania. Ecol Evol. 13:e10160. 10.1002/ece3.10160. PMC10259769 37313272

[arag098-B41] Lüdecke D, Ben-Shachar MS, Patil I, Waggoner P, Makowski D. 2021. Performance: an R package for assessment, comparison and testing of statistical models. J Open Source Softw. 6:3139. 10.21105/joss.03139.

[arag098-B42] Madsen A, de Silva S. 2024. Societies with fission-fusion dynamics as complex adaptive systems: the importance of scale. Philos Trans R Soc Lond B Biol Sci. 379:20230175. 10.1098/rstb.2023.0175. PMC11293855 39034708

[arag098-B43] Mansournia MA, Geroldinger A, Greenland S, Heinze G. 2018. Separation in logistic regression: causes, consequences, and control. Am J Epidemiol. 187:864–870. 10.1093/aje/kwx299. 29020135

[arag098-B44] Markham AC, Gesquiere LR. 2017. Costs and benefits of group living in primates: an energetic perspective. Philos Trans R Soc Lond B Biol Sci. 372:20160239. 10.1098/rstb.2016.0239. PMC5498300 28673916

[arag098-B45] McComb K et al 2011. Leadership in elephants: the adaptive value of age. Proc R Soc Lond B Biol Sci. 278:3270–3276. 10.1098/rspb.2011.0168. PMC3169024 21411454

[arag098-B46] Merkle JA, Fortin D, Morales JM. 2014. A memory-based foraging tactic reveals an adaptive mechanism for restricted space use. Ecol Lett. 17:924–931. 10.1111/ele.12294. 24811575

[arag098-B47] Moss CJ . 1988. Elephant memories: thirteen years in the life of an elephant family. University of Chicago Press.

[arag098-B48] Muller Z . 2018. Population structure of giraffes is affected by management in the Great Rift Valley, Kenya. PLoS One. 13:e0189678. 10.1371/journal.pone.0189678. PMC5751992 29298338

[arag098-B49] Muller Z, Cantor M, Cuthill IC, Harris S. 2018. Giraffe social preferences are context dependent. Anim Behav. 146:37–49. 10.1016/j.anbehav.2018.10.006.

[arag098-B50] Muller Z, Cuthill IC, Harris S. 2019. Giraffe (Giraffa camelopardalis) social networks in areas of contrasting human activity and lion density. Ethology. 125:702–715. 10.1111/eth.12923.

[arag098-B51] Mutinda HS, Poole JH, Moss CJ. 2011. Decision making and leadership in using the ecosystem. In: Moss CJ, Croze H, Lee PC, editors. The Amboseli elephants: a long-term perspective on a long-lived species. University of Chicago Press. p. 246–259.

[arag098-B52] O’connor D et al 2019. Updated geographic range maps for giraffe, Giraffa spp., throughout sub-Saharan Africa, and implications of changing distributions for conservation. Mamm Rev. 49:285–299. 10.1111/mam.12165.

[arag098-B53] Petit O, Gautrais J, Leca JB, Theraulaz G, Deneubourg JL. 2009. Collective decision-making in white-faced capuchin monkeys. Proc Biol Sci. 276:3495–3503. 10.1098/rspb.2009.0983. PMC2817197 19605395

[arag098-B54] Pillot MH, Deneubourg JL. 2010. Collective movements, initiation and stops: diversity of situations and law of parsimony. Behav Processes. 84:657–661. 10.1016/j.beproc.2010.02.024. 20211709

[arag098-B55] Pomeroy D, Matovu B, Kibuule M. 2024. Habituation of large mammals to passing vehicles. Afr J Ecol. 62:e13243. 10.1111/aje.13243.

[arag098-B56] Pontius RG Jr, Parmentier B. 2014. Recommendations for using the relative operating characteristic (ROC). Landsc Ecol. 29:367–382. 10.1007/s10980-013-9984-8.

[arag098-B57] Posit team . 2026. RStudio: integrated development environment for R. Posit Software, PBC, Boston, MA. http://www.posit.co/. [Accessed May 13, 2026].

[arag098-B58] Prehn SG et al 2019. Seasonal variation and stability across years in a social network of wild giraffe. Anim Behav. 157:95–104. 10.1016/j.anbehav.2019.08.018.

[arag098-B59] Pyritz LW, Kappeler PM, Fichtel C. 2011. Coordination of group movements in wild red-fronted lemurs (Eulemur rufifrons): processes and influence of ecological and reproductive seasonality. Folia Primatol (Basel). 32:1325–1347. 10.1007/s10764-011-9549-0. PMC3228953 22207771

[arag098-B60] Ramos A, Petit O, Longour P, Pasquaretta C, Sueur C. 2015. Collective decision-making during group movements in European bison, Bison bonasus. Anim Behav. 109:149–160. 10.1016/j.anbehav.2015.08.016.

[arag098-B61] Ramseyer A, Boissy A, Dumont B, Thierry B. 2009. Decision making in group departures of sheep is a continuous process. Anim Behav. 78:71–78. 10.1016/j.anbehav.2009.03.017.

[arag098-B62] Raveling DG . 1969. Preflight and flight behavior of Canada geese. Auk. 86:671–681. 10.2307/4083454.

[arag098-B63] R Core Team . 2022. R: a language and environment for statistical computing. R Foundation for Statistical Computing. https://www.R-project.org/. [Accessed May 13, 2026].

[arag098-B64] Rieucau G, Giraldeau LA. 2011. Exploring the costs and benefits of social information use: an appraisal of current experimental evidence. Philos Trans R Soc Lond B Biol Sci. 366:949–957. 10.1098/rstb.2010.0325. PMC3049093 21357217

[arag098-B65] Scholes RJ, Archer SR. 1997. Tree-grass interactions in savannas. Ann Rev Ecol Evol Syst. 28:517–544. 10.1146/annurev.ecolsys.28.1.517.

[arag098-B66] Schweinfurth MK, Baldridge DB, Finnerty K, Call J, Knoblich GK. 2022. Inter-individual coordination in walking chimpanzees. Curr Biol. 32:5138–5143.e3. 10.1016/j.cub.2022.09.059. 36270278

[arag098-B67] Seeber PA, Ciofolo I, Ganswindt A. 2012. Behavioural inventory of the giraffe (Giraffa camelopardalis). BMC Res Notes. 5:650. 10.1186/1756-0500-5-650. PMC3599642 23173954

[arag098-B68] Seltmann A, Majolo B, Schülke O, Ostner J. 2013. The organization of collective group movements in wild Barbary Macaques (Macaca sylvanus): social structure drives processes of group coordination in macaques. PLoS One. 8:e67285. 10.1371/journal.pone.0067285. PMC3689719 23805305

[arag098-B69] Seppänen J-T, Forsman JT, Monkkonen M, Thomson RL. 2007. Social information use is a process across time, space, and ecology, reaching heterospecifics. Ecology. 88:1622–1633. 10.1890/06-1757.1. 17645008

[arag098-B70] Strandburg-Peshkin A, Papageorgiou D, Crofoot MC, Farine DR. 2018. Inferring influence and leadership in moving animal groups. Philos Trans R Soc Lond B Biol Sci. 373:20170006. 10.1098/rstb.2017.0006. PMC5882976 29581391

[arag098-B71] Strauss MKL, Kilewo M, Rentsch D, Packer C. 2015. Food supply and poaching limit giraffe abundance in the Serengeti. Popul Ecol. 57:505–516. 10.1007/s10144-015-0499-9.

[arag098-B72] Sueur C, Deneubourg J. 2011. Self-organization in primates: understanding the rules underlying collective movements. Folia Primatol (Basel). 32:1413–1432. 10.1007/s10764-011-9520-0.

[arag098-B73] Sueur C, Deneubourg J-L, Petit O. 2011. From the first intention movement to the last joiner: macaques combine mimetic rules to optimize their collective decisions. Proc Biol Sci. 278:1697–1704. 10.1098/rspb.2010.2084. PMC3081774 21084348

[arag098-B74] Sueur C, Petit O. 2008. Shared or unshared consensus decision in macaques? Behav Processes. 78:84–92. 10.1016/j.beproc.2008.01.004. 18281161

[arag098-B75] Sueur C, Petit O, Deneubourg J-L. 2009. Selective mimetism at departure in collective movements of Macaca tonkeana: an experimental and theoretical approach. Anim Behav. 78:1087–1095. 10.1016/j.anbehav.2009.07.029.

[arag098-B76] Sundaresan SR, Fischhoff IR, Rubenstein DI. 2007. Male harassment influences female movements and associations in Grevy's zebra (Equus grevyi). Behav Ecol. 18:860–865. 10.1093/beheco/arm055.

[arag098-B77] Sutherland C et al 2023. Practical advice on variable selection and reporting using Akaike information criterion. Proc Biol Sci. 290:20231261. 10.1098/rspb.2023.1261. PMC10523071 37752836

[arag098-B78] Therneau T . 2024. A package for survival analysis in R. R package version 3.6-4, [R Package]. https://CRAN.R-project.org/package=survival.

[arag098-B79] Valentini G, Mizumoto N, Pratt SC, Pavlic TP, Walker SI. 2020. Revealing the structure of information flows discriminates similar animal social behaviours. eLife. 9:55395. 10.7554/eLife.55395. PMC7392607 32730203

[arag098-B80] van der Jeugd HP, Prins HHT. 2000. Movements and group structure of giraffe (Giraffa camelopardalis) in Lake Manyara National Park, Tanzania. J Zool. 251:15–21. 10.1111/j.1469-7998.2000.tb00588.x.

[arag098-B81] VanderWaal KL, Wang H, McCowan B, Fushing H, Isbell LA. 2014. Multilevel social organization and space use in reticulated giraffe (Giraffa camelopardalis). Behav Ecol. 25:17–26. 10.1093/beheco/art061.

[arag098-B82] Watts DP . 2000. Mountain gorilla habitat use strategies and group movements. In: Boinski S, Garber PA, editors. On the move: how and why animals travel in groups. University of Chicago Press. p. 351–374.

[arag098-B83] Wielgus E et al 2020. Are fission–fusion dynamics consistent among populations? A large-scale study with Cape buffalo. Ecol Evol. 10:9240–9256. 10.1002/ece3.6608. PMC7487245 32953058

[arag098-B84] Wittemyer G, Polansky L, Douglas-Hamilton I, Getz WM. 2008. Disentangling the effects of forage, social rank, and risk on movement autocorrelation of elephants using Fourier and wavelet analysis. Proc Natl Acad Sci U S A. 105:19108–19113. 10.1073/pnas.0801744105. PMC2614723 19060207

