# Supplementary material for: The socioecological correlates of collective movements in giraffes
Source: Behav Ecol. 2026 Aug 21;37(6):arag098. doi: 10.1093/beheco/arag098 (PMC13635948; doi:10.1093/beheco/arag098)
Supplement: Supplementary file 1 [file arag098_supplementary_data.docx]

**THE SOCIOECOLOGICAL CORRELATES OF COLLECTIVE MOVEMENTS IN GIRAFFES**

**SUPPLEMENTARY MATERIALS**

Table S1: Collinearity diagnostics for logistic regression models predicting movement initiation. Generalized Variance Inflation Factors (GVIF) and tolerance values indicate low multicollinearity among predictors, confirming that parameter estimates are not inflated by shared variance.

| **Term** | **GVIF** | **GVIF 95% CI adj.** | **Adjusted**  **GVIF** | **Tolerance** | **Tolerance 95% CI** |
| --- | --- | --- | --- | --- | --- |
| Habitat type | 1.46 | [1.38, 1.56] | 1.21 | 0.68 | [0.64, 0.72] |
| Group type | 1.41 | [1.34, 1.51] | 1.19 | 0.71 | [0.66, 0.75] |
| Time period | 1.18 | [1.13, 1.25] | 1.09 | 0.85 | [0.80, 0.89] |
| scale(Group size) | 1.18 | [1.12, 1.25] | 1.08 | 0.85 | [0.80, 0.89] |
| scale(Prop. active) | 1.32 | [1.25, 1.40] | 1.15 | 0.76 | [0.71, 0.80] |

Table S2: Collinearity diagnostics for logistic regression model predicting fission outcomes. Generalized Variance Inflation Factors (GVIF) and tolerance values indicate low multicollinearity among predictors, confirming that parameter estimates are not inflated by shared variance.

| **Term** | **GVIF** | **GVIF 95% CI adj.** | **Adjusted**  **GVIF** | **Tolerance** | **Tolerance 95% CI** |
| --- | --- | --- | --- | --- | --- |
| Habitat type | 1.75 | [1.43, 2.29] | 1.32 | 0.57 | [0.44, 0.70] |
| Group type | 2.03 | [1.64, 2.68] | 1.43 | 0.49 | [0.37, 0.61] |
| Time period | 1.17 | [1.05, 1.62] | 1.08 | 0.85 | [0.62, 0.96] |
| scale(Group size) | 1.52 | [1.28, 1.99] | 1.23 | 0.66 | [0.50, 0.78] |
| scale(Prop. active) | 1.26 | [1.10, 1.68] | 1.12 | 0.8 | [0.60, 0.91] |

Figure S1: Distribution of giraffe group sizes observed in Murchison Falls National Park.

Figure S2: ROC curve showing the performance of the GLMM model predicting the initiation of successful group movements.

Figure S3: ROC curve showing the performance of the bias-reduced logistic regression model predicting group fission, after a successful movement initiation.
